# Hulls of Non-Separable Constacyclic Codes over $F_{q}R$ and Their Applications in Constructing New QECCs

**DOI:** 10.3390/e28091028

**Published:** 2026-09-17

**Authors:** Xiying Zheng, Bo Kong, Enbin Zhang

**Affiliations:** 1 Faculty of Engineering, Huanghe Science and Technology College, Zhengzhou 450063, China; 2 School of Statistics and Mathematics, Henan Finance University, Zhengzhou 450046, China; kongbo666@163.com (B.K.); zhangenbin@hafu.edu.cn (E.Z.)

**Keywords:** constacyclic code, hull, quantum error-correcting codes, construction X, 11T71, 94B05, 94B15

## Abstract

Let $R=F_{q}\left[u_{1},u_{2},u_{3}\right]/\langle u_{i}^{2}=u_{i},u_{i}u_{j}=u_{j}u_{i}=0\rangle$ and $F_{q}R=\left\{\left(a,d\right)|a\in F_{q},d\in R\right\}$, where $q=p^{m}$, *p* is a prime, and $m\in N^{+}$. We investigate the algebraic structure of the non-separable constacyclic codes over the mixed-alphabet ring $F_{q}R$ and then determine the generator polynomials and the dimensions of their hulls. As an application, using the construction X, we obtain some new quantum error-correcting codes that are not listed in the online quantum codes database.

## 1. Introduction

Constacyclic codes, as a natural generalization of classical cyclic codes, possess a unique algebraic structure that not only offers significant advantages in coding theory but also ensures inherent compatibility with quantum shift registers, making them a primary resource for constructing quantum error-correcting codes (QECCs). Moreover, constacyclic codes over mixed alphabets have recently attracted research interest due to their ability to enhance coding flexibility and error-correction performance by integrating algebraic structures from different finite fields or rings. In 1997, Rifa and Pujol [1] first introduced translation-invariant propelinear codes over mixed alphabets and revealed their rich algebraic structures. Brouwer et al. [2] established fundamental bounds for mixed binary/ternary codes over $F_{2}^{n_{2}}F_{3}^{n_{3}}$. Borges et al. [3] and Gao et al. [4] respectively identified $Z_{2}$ and $Z_{4}$-double cyclic codes as submodules of $\frac{Z_{2}\left[x\right]}{x^{r}-1}\times \frac{Z_{2}\left[x\right]}{x^{s}-1}$ and $Z_{4}\left[x\right]$-module $\frac{Z_{4}\left[x\right]}{x^{r}-1}\times \frac{Z_{4}\left[x\right]}{x^{s}-1},$ and both gave the polynomial representations, duals, and generator-polynomial relations. Sagar et al. [5] determined the structure of constacyclic codes over $\frac{Z_{2}\left[u\right]}{\langle u^{2}\rangle }\times \frac{Z_{2}\left[u\right]}{\langle u^{3}\rangle }$ and obtained some binary codes with good parameters from these constacyclic codes. Dinh et al. [6] and Ashraf et al. [7] studied the algebraic structure of $F_{q}R$-cyclic codes and $F_{q}R_{1}R_{2}$-cyclic codes, respectively, and presented a construction of QECCs from separable $F_{q}R$-cyclic codes and $F_{q}R_{1}R_{2}$-cyclic codes, respectively. Shukla et al. [8] investigated the algebraic structure of (θ,Θ)-cyclic codes over $F_{q}R$, where $R=F_{q}+uF_{q}+vF_{q}+uvF_{q}$ with $u^{2}=u,v^{2}=v,uv=vu$; $R_{1}=F_{q}+uF_{q}+vF_{q}+uvF_{q}$ with $u^{2}=1,v^{2}=1,uv=vu$; and $R_{2}=F_{q}\left[u,v,w\right]/\langle u^{2}-1,v^{2}-1,w^{2}-1,uv-vu,vw-wv,wu-uw\rangle$ with $u^{2}=1$, $v^{2}=1,w^{2}=1,uv=vu,vw=wv,wu=uw$. All of them constructed some QECCs with good parameters from the respective cyclic codes. Islam and Prakash [9] determined the algebraic structure of $F_{q}R$-additive λ-constacyclic codes of length (n,m) and obtained some new and better QECCs from these codes, where $R=F_{q}\left[u,v\right]/\langle u^{2}-\gamma u,v^{2}-\delta v$, uv=vu=0〉 with $\gamma ,\delta \in F_{q}^{*}$. Dinh et al. [10] and Wang et al. [11] respectively investigated the algebraic structures of $\left(\lambda_{1},\lambda_{2},\lambda_{3}\right)$-constacyclic codes over $F_{q}RS$ (where $R=F_{q}+uF_{q}$ with $u^{2}=u$ and $S=F_{q}+uF_{q}+vF_{q}$ with $u^{2}=u,v^{2}=v,uv=vu=0$) and $\left(u_{1},u_{2}\right)$-constacyclic codes over $F_{q^{2}}\times \left(F_{q^{2}}+vF_{q^{2}}\right)$, and both constructed new QECCs with better parameters from the respective constacyclic codes.

QECCs are the foundation for realizing fault-tolerant quantum computation and quantum communication. The standard constructions, namely the CSS [12,13] and Hermitian [14] methods, require linear codes to satisfy the dual-containing condition $C^{⊥}\subseteq C$. To overcome the dual-containing restriction, Lisoněk and Singh [15] introduced the quantum construction X over $F_{4}$ by computing the dimension of $C\cap C^{⊥}$, and Degwekar et al. [16] later extended it to $F_{q^{2}}$. The intersection $C\cap C^{⊥}$ is called the hull of a linear code, a concept first introduced by Assmus and Key [17]. Thus, the hull provides a natural tool for constructing QECCs without imposing the dual-containing condition, which is the primary motivation for studying hulls in the context of quantum error correction.

Subsequently, the dimensions of the hulls were determined for general linear codes [18] and for cyclic codes [19,20,21]. Following these foundational results, hulls of more specialized code families have been extensively studied. Gao et al. [22] investigated the hulls of non-separable double cyclic codes over $Z_{2}$ and they also explored the construction of EAQECCs by the hulls of non-separable double cyclic codes. Li et al. [23] investigate the hulls of non-separable double cyclic codes over $Z_{4}$, focusing on the structural properties and dimensions of their hulls. Li et al. [24] studied Hermitian dual-containing constacyclic codes over finite fields and constructed entanglement-assisted QECCs via the Hermitian construction, while Yadav et al. [25] investigated Hermitian hulls of constacyclic codes over non-chain rings and derived new QECCs using the same method. Tian et al. [26] studied hulls of constacyclic codes over finite non-chain rings and constructed new QECCs using the quantum construction X method. Zhang et al. [27] further extended this line of research to Galois hulls over a finite non-chain ring and obtained new QECCs. Hu and Liu [28] determined hulls of cyclic codes over the mixed alphabet $F_{2}\times \left(F_{2}+vF_{2}\right)$ and constructed new QECCs via the quantum construction X method.

Although constacyclic codes over various mixed-alphabet rings have been studied in [5,9,10,11], the hulls of non-separable constacyclic codes over mixed-alphabet rings have not been investigated. Our work therefore extends the theory of separable cyclic codes over mixed alphabets [6,7], the hull theory of non-separable double cyclic codes over $Z_{2}$ and $Z_{4}$ [22,23], the hull theory of constacyclic codes over finite fields and finite rings [24,25,26,27], and the hull theory of cyclic codes over mixed alphabets [28] to the hulls of non-separable constacyclic codes over a new mixed-alphabet ring $F_{q}R$; moreover, we generalize the study of their hulls to this setting and construct new QECCs via the quantum construction X.

The rest of this paper is organized as follows. In Section 2, we introduce the necessary definitions and notations and give some properties of constacyclic codes over R. In Section 3, we present the algebraic structure of constacyclic codes over $F_{q}R$. In Section 4, we study the hulls of non-separable constacyclic codes over $F_{q}R$. In Section 5, via the quantum construction X, we obtain some new QECCs that are better than the best known codes listed in the online quantum codes database [29].

## 2. Preliminaries

Let $F_{q}$ be a finite field, and $F_{q}^{s}$ be an *s*-dimensional subspace over $F_{q}$, where $q=p^{m}$, *p* is a prime, and $m\in N^{+}$. Let λ be a unit in $F_{q}$; for any $c=\left(c_{0},c_{1},\cdots ,c_{s-1}\right)\in F_{q}^{s}$, we define a λ-constacyclic shift $\sigma_{\lambda }$ of c by $\sigma_{\lambda }\left(c\right)=\left(\lambda c_{s-1},c_{0},\cdots ,c_{s-2}\right)$. A linear code *C* over $F_{q}$ is termed a λ-constacyclic code when $\sigma_{\lambda }\left(C\right)=C$. In particular, if λ=1, then *C* is a cyclic code, whereas if λ=−1, *C* is a negacyclic code.

If there exists the bijective correspondence between each codeword $\left(c_{0},c_{1},\cdots ,c_{s-1}\right)\in F_{q}^{s}$ and a polynomial $c_{0}+c_{1}x+\cdots +c_{s-1}x^{s-1}\in F_{q}\left[x\right]/\langle x^{s}-\lambda \rangle$, a λ-constacyclic code of length *s* over $F_{q}$ can be characterized as an ideal of $F_{q}\left[x\right]/\langle x^{s}-\lambda \rangle$.

Let $f\left(x\right)=a_{0}+a_{1}x+a_{2}x^{2}+\cdots +a_{r-1}x^{r-1}+a_{r}x^{r}$ be a polynomial with $a_{0}\neq 0$; the reciprocal polynomial of f(x) is denoted by $f^{*}\left(x\right)=a_{0}^{-1}x^{r}f\left(x^{-1}\right)$.

**Definition** **1.** *Let $c=(c_{0},c_{1},\cdots ,c_{s-1})$, $\tilde{c}=\left({\tilde{c}}_{0},{\tilde{c}}_{1},\cdots ,{\tilde{c}}_{s-1}\right)\in F_{q}^{s}$; the inner product of $c,\tilde{c}$ is defined as*

$$c\cdot \tilde{c}=\sum_{i=0}^{s-1}c_{i}{\tilde{c}}_{i}.$$

**Definition** **2.** *For any code C over $F_{q}$, its Euclidean dual code is defined as*

$$C^{⊥}=\left\{x|y\cdot x=0,\forall \;y\in C\right\}.$$

By Definition 2, we say that *C* is self-orthogonal if $C\subseteq C^{⊥}$, dual-containing if $C^{⊥}\subseteq C$, and self-dual if $C=C^{⊥}$.

**Definition** **3.** *For any code C over $F_{q}$, the hull of C is defined by*

$$\mathrm{Hull}\left(C\right)=C\cap C^{⊥}.$$

Clearly, if Hull(C)=C, then *C* is self-orthogonal; if $\mathrm{Hull}\left(C\right)=C^{⊥}$, then *C* is dual-containing.

Let

$$R=F_{q}\left[u_{1},u_{2},u_{3}\right]/\langle u_{i}^{2}=u_{i},u_{i}u_{j}=u_{j}u_{i}=0\rangle ,$$

where i,j=1,2,3.

Suppose $e_{1}=u_{1},e_{2}=u_{2},e_{3}=u_{3},e_{4}=1-u_{1}-u_{2}-u_{3}$; we can have $e_{1}+e_{2}+e_{3}+e_{4}=1$, $e_{i}^{2}=e_{i}$, and $e_{i}e_{j}=0$ (i≠j), where i,j=1,2,3,4. By the Chinese Remainder Theorem, we have

$$R={⊕}_{i=1}^{4}e_{i}R={⊕}_{i=1}^{4}e_{i}F_{q}.$$

For any d∈R, then *d* can be uniquely written as

$$d=\sum_{i=1}^{4}e_{i}d_{i},$$

where $d_{i}\in F_{q}$ for i=1,2,3,4.

Define a Gray map ϕ as

$$\begin{array}{cc} \; & \phi :R\to F_{q}^{4},\\ \; & d=\sum_{i=1}^{4}d_{i}e_{i}\mapsto \left(d_{1},d_{2},d_{3},d_{4}\right). \end{array}$$

Extend ϕ as

$$\begin{array}{cc} \; & \phi :R^{t}\to F_{q}^{4t},\\ \; & \left(d_{0},d_{1},\cdots ,d_{t-1}\right)\mapsto \left(d_{1,0},\cdots ,d_{1,t-1},d_{2,0},\cdots ,d_{2,t-1},d_{3,0},\cdots ,d_{3,t-1},d_{4,0},\cdots ,d_{4,t-1}\right), \end{array}$$

where $d_{i}=d_{1,i}e_{1}+d_{2,i}e_{2}+d_{3,i}e_{3}+d_{4,i}e_{4}\in R$ for i=0,1,⋯,t−1.

**Definition** **4.** *For any element $c=\left(c_{1},c_{2},\cdots ,c_{t}\right)\in R^{t}$, the Gray weight of c is defined as*

$$w_{G}\left(c\right)=w_{H}\left(\phi \left(c\right)\right),$$

*and the Gray distance of c,d is defined as*

$$d_{G}\left(c,d\right)=w_{G}\left(c-d\right)=w_{H}\left(\phi \left(c-d\right)\right).$$

Let $C_{t}$ be a linear code of length *t* over R; we define

$$\begin{array}{cc} & C_{t,1}=\left\{c_{1}\in F_{q}^{t}|\sum_{i=1}^{4}e_{i}c_{i}\in C_{t},\exists c_{2},c_{3},c_{4}\in F_{q}^{t}\right\},\\ & C_{t,2}=\left\{c_{2}\in F_{q}^{t}|\sum_{i=1}^{4}e_{i}c_{i}\in C_{t},\exists c_{1},c_{3},c_{4}\in F_{q}^{t}\right\},\\ & C_{t,3}=\left\{c_{3}\in F_{q}^{t}|\sum_{i=1}^{4}e_{i}c_{i}\in C_{t},\exists c_{1},c_{2},c_{4}\in F_{q}^{t}\right\},\\ & C_{t,4}=\left\{c_{4}\in F_{q}^{t}|\sum_{i=1}^{4}e_{i}c_{i}\in C_{t},\exists c_{1},c_{2},c_{3}\in F_{q}^{t}\right\}. \end{array}$$

Therefore, $C_{t,1},C_{t,2},C_{t,3},C_{t,4}$ are linear codes of length *t* over $F_{q}$, and

$$C_{t}={⊕}_{i=1}^{4}e_{i}C_{t,i}.$$

Based on the Gray map ϕ, Definition 4, and the lemma and theorems in [30], we have the following properties of constacyclic codes over R.

**Lemma** **1.** *ϕ is a distance-preserving linear bijection from $R^{t}$ to $F_{q}^{4t}$.*

**Proof.**  The proof of this theorem is identical to that of Lemma 1 in [30], and thus is omitted here. □

**Theorem** **1.** *Let $C_{t}={⨁}_{i=1}^{4}e_{i}C_{t,i}$ be a λ-constacyclic code of length t over R. Then,*

$$C_{t}=\langle \sum_{i=1}^{4}e_{i}g_{i}\left(x\right)\rangle ,$$

*where $\lambda =\sum_{i=1}^{4}\lambda_{i}e_{i}$, and $g_{i}\left(x\right)$ is the generator polynomial of $C_{t,i}$ for i=1,2,3,4.*

**Proof.**  The proof of this theorem is identical to that of Theorem 2 in [30], and thus is omitted here. □

**Theorem** **2.** *Let $C_{t}={⨁}_{i=1}^{4}e_{i}C_{t,i}$ be a λ-constacyclic code of length t over R. Then,*

$$C_{t}^{⊥}=\langle \sum_{i=1}^{4}e_{i}g_{i}^{*}\left(x\right)\rangle ,$$

where $\lambda =\sum_{i=1}^{4}\lambda_{i}e_{i}$, $g_{i}\left(x\right)$ is the generator polynomial of $C_{t,i}$, $g_{i}^{*}\left(x\right)$ is the reciprocal polynomial of $g_{i}\left(x\right)$, and i=1,2,3,4.

**Proof.**  The proof of this theorem is identical to that of Theorem 4 in [30], and thus is omitted here. □

## 3. Constacyclic Codes over $F_{q}R$

In this section, we will define a mixed-alphabet ring $F_{q}R$ and give some basic concepts and properties of constacyclic codes over $F_{q}R$.

Let

$$R_{s,t}=\frac{F_{q}\left[x\right]}{\langle x^{s}-\lambda_{1}\rangle }\times \frac{R[x]}{\langle x^{t}-\lambda_{2}\rangle }$$

and

$$F_{q}R=\left\{\left(c,d\right)|c\in F_{q},d\in R\right\}.$$

The addition (+) and multiplication (×) on the set $F_{q}R$ are defined respectively as follows. For any $\left(c_{1},d_{1}\right),\left(c_{2},d_{2}\right)\in F_{q}R$; then,

$$\begin{array}{cc} \; & \left(c_{1},d_{1}\right)+\left(c_{2},d_{2}\right)=\left(c_{1}+c_{2},d_{1}+d_{2}\right),\\ \; & \left(c_{1},d_{1}\right)\times \left(c_{2},d_{2}\right)=\left(c_{1}c_{2},d_{1}d_{2}\right). \end{array}$$

With this typical definition, the set $F_{q}R$ forms a ring.

For any element $r=r_{0}+r_{1}u_{1}+r_{2}u_{2}+r_{3}u_{3}\in R$, a ring homomorphism ϱ is defined as

$$\begin{array}{cc} \; & \varrho :R\to F_{q},\\ \; & r=r_{0}+r_{1}u_{1}+r_{2}u_{2}+r_{3}u_{3}\mapsto r_{0}. \end{array}$$

For any element r∈R, an R-scalar multiplication • is defined as

$$\begin{array}{cc} \; & •:R\times F_{q}R\to F_{q}R,\\ \; & r•(c,d)\mapsto (\varrho (r)c,rd). \end{array}$$

We extend the multiplication • as

$$\begin{array}{cc} \; & •:R\times F_{q}^{s}\times R^{t}\to F_{q}^{s}\times R^{t},\\ \; & r•\left(c_{0},c_{1},\cdots ,c_{s-1},d_{0},d_{1},\cdots ,d_{t-1}\right)\mapsto \left(\varrho \left(r\right)c_{0},\varrho \left(r\right)c_{1},\cdots ,\varrho \left(r\right)c_{s-1},rd_{0},rd_{1},\cdots ,rd_{t-1}\right). \end{array}$$

From this multiplication, we can get that $F_{q}^{s}\times R^{t}$ is an R-module.

**Definition** **5.** *Let C be a non-empty subset of $F_{q}^{s}\times R^{t}$. If C is an R-module, we say that C is an $F_{q}R$-linear code of block length (s,t).*

Throughout this paper, let $\alpha =(\lambda_{1},\lambda_{2})$ be a unit element of $F_{q}R$, where $\lambda_{1}$ is a unit of $F_{q}$ and $\lambda_{2}=\sum_{i=1}^{4}\lambda_{2,i}e_{i}$ is a unit of R. Let *C* be an $F_{q}R$-linear code of block length (s,t); for any $c=(c_{0},c_{1},\cdots ,c_{s-1},d_{0},d_{1},\cdots ,d_{t-1})\in C$, the α-constacyclic shift $\sigma_{\alpha }$ of c is defined as $\sigma_{\alpha }\left(c\right)=\left(\lambda_{1}c_{s-1},c_{0},\cdots ,c_{s-2},\lambda_{2}d_{t-1},d_{0},\cdots ,d_{t-2}\right)$. An $F_{q}R$-linear code *C* of block length (s,t) is called an $F_{q}R$-α-constacyclic code if $\sigma_{\alpha }\left(C\right)=C$. In particular, if α=(1,1), then *C* is an $F_{q}R$-cyclic code. Meanwhile, if α=(−1,−1), *C* is an $F_{q}R$-negacyclic code.

Each codeword $\left(c_{0},c_{1},\cdots ,c_{s-1},d_{0},d_{1},\cdots ,d_{t-1}\right)\in F_{q}^{s}\times R^{t}$ corresponds bijectively to a polynomial $r\left(x\right)=\left(c_{0}+c_{1}x+\cdots +c_{s-1}x^{s-1},d_{0}+d_{1}x+\cdots +d_{t-1}x^{t-1}\right)\in R_{s,t}$; we denote r(x)=(c(x),b(x)).

For any element $f\left(x\right)=r_{0}+r_{1}x+\cdots +r_{n-1}x^{n-1}\in R\left[x\right]$ with the element r(x)=(c(x),d(x)), we define a multiplication as follows

f(x)★r(x)=f(x)★(c(x),d(x))=(ϱ(f(x))c(x),f(x)d(x)),

where $\varrho \left(f\left(x\right)\right)=\varrho \left(r_{0}\right)+\varrho \left(r_{1}\right)x+\cdots +\varrho \left(r_{n-1}\right)x^{n-1}$. From this multiplication, we can get that $R_{s,t}$ is an R[x]-module.

**Theorem** **3.** *A linear code C of block length (s,t) over $F_{q}R$ is an $F_{q}R$-α-constacyclic code if and only if C is an R[x]-submodule of $R_{s,t}$, where $\alpha =(\lambda_{1},\lambda_{2})$.*

**Proof.**  The proof of this theorem is identical to that of Theorem 2.6 in [10], and thus is omitted here. □

We define a Gray map ψ as

$$\begin{array}{cc} \; & \psi :F_{q}R\to F_{q}^{5},\\ \; & \left(c,d\right)=\left(c,\sum_{i=1}^{4}d_{i}e_{i}\right)\mapsto \left(c,d_{1},d_{2},d_{3},d_{4}\right). \end{array}$$

We extend ψ as

$$\begin{array}{cc} \; & \psi :F_{q}^{s}\times R^{t}\to F_{q}^{s+4t},\\ \; & (c_{0},c_{1},\cdots ,c_{s-1},d_{0},d_{1},\cdots ,d_{t-1})\mapsto \\ \; & (c_{0},c_{1},\cdots ,c_{s-1},d_{1,0},\cdots ,d_{1,t-1},d_{2,0},\cdots ,d_{2,t-1},d_{3,0},\cdots ,d_{3,t-1},d_{4,0},\cdots ,d_{4,t-1}), \end{array}$$

where $d_{i}=d_{1,i}e_{1}+d_{2,i}e_{2}+d_{3,i}e_{3}+d_{4,i}e_{4}\in R$ for i=0,1,⋯,t−1.

**Definition** **6.** *For any element $\left(c,d\right)=\left(c_{0},c_{1},\cdots ,c_{s-1},d_{0},d_{1},\cdots ,d_{t-1}\right)\in F_{q}^{s}\times R^{t}$, the Gray weight of (c,d) is defined as*

$$w_{G}\left(\left(c,d\right)\right)=w_{H}\left(c\right)+w_{G}\left(d\right),$$

*and the Gray distance between (c,d) and $\left(\tilde{c},\tilde{d}\right)$ is defined as*

$$d_{G}\left(\left(c,d\right),\left(\tilde{c},\tilde{d}\right)\right)=w_{H}(\left(c-\tilde{c}\right)+w_{H}\left(\phi \left(d-\tilde{d}\right)\right).$$

**Lemma** **2.** *ψ is a distance-preserving linear bijection from $F_{q}^{s}\times R^{t}$ to $F_{q}^{s+4t}$.*

**Proof.**  For any $a=(a_{0},a_{1},\cdots ,a_{s-1},d_{0},d_{1},\cdots ,d_{t-1})$, $\tilde{a}=\left(a_{0}^{\prime },a_{1}^{\prime },\cdots ,a_{s-1}^{\prime },d_{0}^{\prime },d_{1}^{\prime },\cdots ,d_{t-1}^{\prime }\right)\in F_{q}^{s}\times R^{t}$, where $d_{i}=d_{1,i}e_{1}+d_{2,i}e_{2}+d_{3,i}e_{3}+d_{4,i}e_{4},{\tilde{d}}_{i}=d_{1,i}^{\prime }e_{1}+d_{2,i}^{\prime }e_{2}+d_{3,i}^{\prime }e_{3}+d_{4,i}^{\prime }e_{4}\in R$, i=0,1,⋯,t−1. Then,

$$\begin{array}{cc} \psi (a+\tilde{a}) & =(a_{0}+a_{0}^{\prime },a_{1}+a_{1}^{\prime },\cdots ,a_{s-1}+a_{s-1}^{\prime },\\ \; & \;\;\;\;d_{1,0}+d_{1,0}^{\prime },\cdots ,d_{1,t-1}+d_{1,t-1}^{\prime },d_{2,0}+d_{2,0}^{\prime },\cdots ,d_{2,t-1}+d_{2,t-1}^{\prime },\\ \; & \;\;\;\;d_{3,0}+d_{3,0}^{\prime },\cdots ,d_{3,t-1}+d_{3,t-1}^{\prime },d_{4,0}+d_{4,0}^{\prime },\cdots ,d_{4,t-1}+d_{4,t-1}^{\prime })\\ \; & =\psi \left(a\right)+\psi \left(\tilde{a}\right), \end{array}$$

and

$$\begin{array}{cc} \psi (ka) & =\psi (ka_{0},ka_{1},\cdots ,ka_{s-1},kd_{0},kd_{1},\cdots ,kd_{t-1})\\ \; & =(ka_{0},ka_{1},\cdots ,ka_{s-1},kd_{1,0},\cdots ,kd_{1,t-1},kd_{2,0},\cdots ,kd_{2,t-1},\\ \; & \;\;\;kd_{3,0},\cdots ,kd_{3,t-1},kd_{4,0},\cdots ,kd_{4,t-1})\\ \; & =k\psi (a). \end{array}$$

So ψ is a linear map.

Let

$$\psi \left(a\right)=\psi \left(\tilde{a}\right),$$

Then,

$$\begin{array}{cc} \; & \left(a_{0},a_{1},\cdots ,a_{s-1},d_{1,0},\cdots ,d_{1,t-1},d_{2,0},\cdots ,d_{2,t-1},d_{3,0},\cdots ,d_{3,t-1},d_{4,0},\cdots ,d_{4,t-1}\right)\\ \; & =(a_{0}^{\prime },a_{1}^{\prime },\cdots ,a_{s-1}^{\prime },d_{1,0}^{\prime },\cdots ,d_{1,t-1}^{\prime },d_{2,0}^{\prime },\cdots ,d_{2,t-1}^{\prime },d_{3,0}^{\prime },\cdots ,d_{3,t-1}^{\prime },d_{4,0}^{\prime },\cdots ,d_{4,t-1}^{\prime }), \end{array}$$

We have

$$a=\tilde{a}.$$

So ψ is a injective.

Since

$$\left|F_{q}^{s}\times R^{t}\right|=q^{s+4t}=\left|F_{q}^{s+4t}\right|,$$

ψ is a bijection.

Since

$$\psi \left(a-\tilde{a}\right)=\psi \left(a\right)-\psi \left(\tilde{a}\right)$$

and

$$\begin{array}{cc} d_{G}\left(a,\tilde{a}\right) & =w_{H}\left(a_{0}-a_{0}^{\prime },a_{1}-a_{1}^{\prime },\cdots ,a_{s-1}-a_{s-1}^{\prime }\right)\\ \; & \;\;\;+w_{H}\left(\phi \left(d_{0}-d_{0}^{\prime },d_{1}-d_{1}^{\prime },\cdots ,d_{t-1}-d_{t-1}^{\prime }\right)\right)\\ \; & =w_{H}(\psi \left(\left(a-\tilde{a}\right)\right)=w_{H}\left(\psi \left(a\right)-\psi \left(\tilde{a}\right)\right)\\ \; & =d_{H}\left(\psi \left(a\right),\psi \left(\tilde{a}\right)\right). \end{array}$$

ψ is a distance-preserving linear bijection from $F_{q}^{s}\times R^{t}$ to $F_{q}^{s+4t}$. □

For any element $\left(c,d\right)=\left(e_{1}c+e_{2}c+e_{3}c+e_{4}c,e_{1}d_{1}+e_{2}d_{2}+e_{3}d_{3}+e_{4}d_{4}\right)\in F_{q}^{s}\times R^{t}$, where $c\in F_{q}^{s},d=e_{1}d_{1}+e_{2}d_{2}+e_{3}d_{3}+e_{4}d_{4}\in R^{t}$, we define

$$\begin{array}{cc} & C_{1}=\left\{\left(c,d_{1}\right)\in F_{q}^{s}\times F_{q}^{t}|a\in F_{q}^{s},d_{1}\in C_{t,1}\right\},\\ & C_{2}=\left\{\left(c,d_{2}\right)\in F_{q}^{s}\times F_{q}^{t}|a\in F_{q}^{s},d_{2}\in C_{t,2}\right\},\\ & C_{3}=\left\{\left(c,d_{3}\right)\in F_{q}^{s}\times F_{q}^{t}|a\in F_{q}^{s},d_{3}\in C_{t,3}\right\},\\ & C_{4}=\left\{\left(c,d_{4}\right)\in F_{q}^{s}\times F_{q}^{t}|a\in F_{q}^{s},d_{4}\in C_{t,4}\right\}. \end{array}$$

Clearly, $C_{1},C_{2},C_{3},C_{4}$ are linear codes of block length (s,t) over $F_{q}$, and

$$C={⊕}_{i=1}^{4}e_{i}C_{i}.$$

**Definition** **7.** *Let $p_{1}\left(x\right)=\left(a_{1}\left(x\right),b_{1}\left(x\right)\right),p_{2}\left(x\right)=\left(a_{2}\left(x\right),b_{2}\left(x\right)\right)$ be two elements of $R_{s,t}$. Define the map*

$$\begin{array}{cc} \; & \circ :R_{s,t}\times R_{s,t}\to R\left[x\right]/\left(x^{l}-\lambda_{2}\right) \end{array}$$

*such that*

$$\begin{array}{cc} \circ (p_{1}\left(x\right),p_{2}\left(x\right))\equiv & e_{1}a_{1}\left(x\right)a_{2}^{*}\left(x\right)x^{l-\deg(a_{2}\left(x\right))-1}\frac{x^{l}-\lambda_{2}}{x^{s}-\lambda_{1}}\\ \; & +b_{1}\left(x\right)b_{2}^{*}\left(x\right)x^{l-\deg(b_{2}\left(x\right))-1}\frac{x^{l}-\lambda_{2}}{x^{t}-\lambda_{2}}\left(\mathrm{mod}\left(x^{l}-\lambda_{2}\right)\right), \end{array}$$

*where l=lcm(s,t).*

The map ∘ is a bilinear map between R[x]-modules. For more details on Definition 7, see Definition 21 in [6]. For convenience, we denote $\circ (p_{1}\left(x\right),p_{2}\left(x\right))$ by $p_{1}\left(x\right)\circ p_{2}\left(x\right)$.

**Theorem** **4.** *Let C be an $F_{q}R$-α-constacyclic code of block length (s,t), where $\alpha =(\lambda_{1},\lambda_{2})$. Then,*

C=〈(f(x),0),(l(x),g(x))〉,

*where $f\left(x\right),l\left(x\right)\in \frac{F_{q}\left[x\right]}{\langle x^{s}-\lambda_{1}\rangle }$, $f\left(x\right)|\left(x^{s}-\lambda_{1}\right)$, $g\left(x\right)\in \frac{R[x]}{\langle x^{t}-\lambda_{2}\rangle }$, $g\left(x\right)|\left(x^{t}-\lambda_{2}\right)$, $g\left(x\right)=\sum_{i=1}^{4}e_{i}g_{i}\left(x\right)$, and $g_{i}\left(x\right)|\left(x^{t}-\lambda_{2i}\right)$ with $g_{i}\left(x\right)\in F_{q}\left[x\right]$ for i=1,2,3,4.*

**Proof.**  The proof of this theorem is identical to that of Theorem 9 in [6], and thus is omitted here. □

**Theorem** **5.** *Let C=〈(f(x),0),(l(x),g(x))〉 be an $F_{q}R$-α-constacyclic code of block length (s,t), where $\alpha =(\lambda_{1},\lambda_{2})$, $f\left(x\right)k\left(x\right)=x^{s}-\lambda_{1}$, $g\left(x\right)=\sum_{i=1}^{4}e_{i}g_{i}\left(x\right)$ with $g_{i}\left(x\right)h_{i}\left(x\right)=x^{t}-\lambda_{2i}$ and $f\left(x\right)|h_{1}\left(x\right)l\left(x\right)$, $g_{i}\left(x\right)\in F_{q}\left[x\right]$ for i=1,2,3,4. Then, C has $q^{k}$ codewords, where $k=\deg\left(k\left(x\right)\right)+\Sigma_{i=1}^{4}\deg\left(h_{i}\left(x\right)\right)$.*

**Proof.**  The proof of this theorem is identical to that of Theorem 17 in [6], and thus is omitted here. □

**Definition** **8.** *Let C be an $F_{q}R$-α-constacyclic code of block length (s,t), where $\alpha =(\lambda_{1},\lambda_{2})$. Let $C_{s}$ and $C_{t}$ denote the projections of C onto the first s coordinates and the last t coordinates, respectively. The code C is called separable if*

$$C=C_{s}\times C_{t}.$$

By the same method as Lemma 13 in [6], we can obtain the following lemma.

**Lemma** **3.** *Let C=〈(f(x),0),(l(x),g(x))〉 be an $F_{q}R$-α-constacyclic code of block length (s,t), where $\alpha =(\lambda_{1},\lambda_{2})$. Then,*

$$C_{s}=\langle \gcd\left(f\left(x\right),l\left(x\right)\right)\rangle$$

*and*

$$C_{t}=\langle g\left(x\right)\rangle .$$

By the same method as Theorem 15 in [6], we can obtain the following corollary.

**Corollary** **1.** *Let C=〈(f(x),0),(l(x),g(x))〉 be an $F_{q}R$-α-constacyclic code of block length (s,t), where $\alpha =(\lambda_{1},\lambda_{2})$. Then, C is separable if and only if l(x)=0.*

**Definition** **9.** *Let $r=(c_{0},c_{1},\cdots ,c_{s-1},d_{0},d_{1},\cdots ,d_{t-1})$, $\tilde{r}=\left({\tilde{c}}_{0},{\tilde{c}}_{1},\cdots ,{\tilde{c}}_{s-1},{\tilde{d}}_{0},{\tilde{d}}_{1},\cdots ,\;{\tilde{d}}_{t-1}\right)\in F_{q}^{s}\times R^{t}$; the inner product of $r,\;\tilde{r}$, is defined as*

$$r\cdot \tilde{r}=e_{1}\sum_{i=0}^{s-1}c_{i}{\tilde{c}}_{i}+\sum_{j=0}^{t-1}d_{j}{\tilde{d}}_{j}.$$

**Lemma** **4.** *Let C be an $F_{q}R$-α-constacyclic code of block length (s,t), where $\alpha =(\lambda_{1},\lambda_{2})$. Then, $C^{⊥}$ is an $F_{q}R$-$\alpha^{-1}$-constacyclic code of block length (s,t), where $\alpha^{-1}=\left(\lambda_{1}^{-1},\lambda_{2}^{-1}\right)$.*

**Proof.**  Suppose the order of $\lambda_{1}$ in $F_{q}$ is *a* and the order of $\lambda_{2}$ in R is *b*; l=lcm(s,t). For any $r=\left(c_{0},c_{1},\cdots ,c_{s-1},d_{0},d_{1},\cdots ,d_{t-1}\right)\in C^{⊥}$ and $\tilde{r}=\left({\tilde{c}}_{0},{\tilde{c}}_{1},\cdots ,{\tilde{c}}_{s-1},{\tilde{d}}_{0},{\tilde{d}}_{1},\cdots ,{\tilde{d}}_{t-1}\right)\in C$, we have

$$\sigma_{\alpha }^{abl-1}\left(\tilde{r}\right)=\left({\tilde{c}}_{1},\cdots ,{\tilde{c}}_{s-1},\lambda_{1}^{-1}{\tilde{c}}_{0},{\tilde{d}}_{1},\cdots ,{\tilde{d}}_{t-1},\lambda_{2}^{-1}{\tilde{d}}_{0}\right)\in C.$$

So

$$\begin{array}{cc} 0 & =\sigma_{\alpha }^{abl-1}\left(\tilde{r}\right)\cdot r\\ \; & =e_{1}\left(c_{0}{\tilde{c}}_{1}+\cdots +c_{s-2}{\tilde{c}}_{s-1}+\lambda_{1}^{-1}c_{s-1}{\tilde{c}}_{0}\right)+\left(d_{0}{\tilde{d}}_{1}+\cdots +d_{t-2}{\tilde{d}}_{t-1}+\lambda_{2}^{-1}d_{t-1}{\tilde{d}}_{0}\right). \end{array}$$

We can have

$$\begin{array}{cc} \sigma_{\alpha^{-1}}\left(r\right)\cdot \tilde{r} & =e_{1}\left(\lambda_{1}^{-1}c_{s-1}{\tilde{c}}_{0}+c_{0}{\tilde{c}}_{1}+\cdots +c_{s-2}{\tilde{c}}_{s-1}\right)+\lambda_{2}^{-1}d_{t-1}{\tilde{d}}_{0}+d_{0}{\tilde{d}}_{1}+\cdots +d_{t-2}{\tilde{d}}_{t-1}\\ \; & =0. \end{array}$$

This implies

$$\sigma_{\alpha^{-1}}\left(r\right)\in C^{⊥},$$

so $C^{⊥}$ is an $F_{q}R$-$\alpha^{-1}$-constacyclic code of block length (s,t). □

**Theorem** **6.** *Let C=〈(f(x),0),(l(x),g(x))〉 be an $F_{q}R$-$\left(\lambda_{1},\lambda_{2}\right)$-constacyclic code of block length (s,t), where $g\left(x\right)=\sum_{i=1}^{4}e_{i}g_{i}\left(x\right)$. Then, $C^{⊥}=\langle \left(\tilde{f}\left(x\right),0\right),\left(\tilde{l}\left(x\right),\tilde{g}\left(x\right)\right)\rangle$, where*

$$\begin{array}{cc} \; & \tilde{f}\left(x\right)={\left(\frac{x^{s}-\lambda_{1}}{\gcd(f(x),l(x))}\right)}^{*},\tilde{l}\left(x\right)=\frac{x^{s}-\lambda_{1}}{f^{*}\left(x\right)}\mu \left(x\right),\\ \; & \mu \left(x\right)\equiv -x^{\mathrm{lcm}\left(s,t\right)-\deg\left(g_{1}\left(x\right)\right)+\deg\left(l\left(x\right)\right)}{\left(\frac{l^{*}\left(x\right)}{\gcd{\left(f\left(x\right),l\left(x\right)\right)}^{*}}\right)}^{-1}\left(\mathrm{mod}\frac{f^{*}\left(x\right)}{\gcd{\left(f\left(x\right),l\left(x\right)\right)}^{*}}\right),\\ \; & \tilde{g}\left(x\right)=\sum_{i=1}^{4}e_{i}{\tilde{g}}_{i}\left(x\right),{\tilde{g}}_{1}\left(x\right)=\frac{\left(x^{t}-\lambda_{21}\right)\gcd{\left(f\left(x\right),l\left(x\right)\right)}^{*}}{f^{*}\left(x\right)g_{1}^{*}\left(x\right)},{\tilde{g}}_{i}\left(x\right)={\left(\frac{x^{t}-\lambda_{2i}}{g_{i}\left(x\right)}\right)}^{*}for\;i=2,3,4, \end{array}$$

**Proof.**  The proof of this theorem is identical to that of Theorem 31 in [6], and thus is omitted here. □

## 4. Hulls of Non-Separable Constacyclic Codes over $F_{q}R$

In this section, we focus on non-separable constacyclic codes over the mixed-alphabet ring $F_{q}R$ rather than separable constacyclic codes. A separable code over $F_{q}R$ is merely a direct product of component codes, so its components are completely decoupled and its parameters are essentially determined by those of the individual components. In contrast, a non-separable code is an arbitrary submodule of the ambient mixed-alphabet module, which allows non-trivial algebraic coupling among the different alphabet components. This richer structure typically yields larger minimum distances and better parameters, and it is precisely this additional freedom that enables us to construct new QECCs with improved parameters via the quantum construction X.

Accordingly, we will determine the generator polynomials and the dimensions of the hulls of non-separable constacyclic codes over $F_{q}R$. Throughout this section, we always assume that *s* and *t* are coprime odd positive integers and that l=lcm(s,t).

**Lemma** **5** ([19])**.** *Let C=〈f(x)〉 and D=〈g(x)〉 be λ-constacyclic codes of length n over $F_{q}$; f(x) and g(x) are generator polynomials of C and D, respectively. Then, C∩D is a λ-constacyclic code generated by lcm(f(x),g(x)).*

By the same method of Lemma 4.2 in [22], we can have the following lemma.

**Lemma** **6.** *Let C=〈(f(x),0),(l(x),g(x)〉 be a non-separable $F_{q}R$-$\left(\lambda_{1},\lambda_{2}\right)$-constacyclic code of block length (s,t).*

*1.*  *If $\lambda_{1}=\lambda_{2}=1$, then $C=\langle (\left(x-1)f_{1}\left(x\right),0\right),\left(f_{1}\left(x\right),g\left(x\right)\right)\rangle$, where $f_{1}\left(x\right)|\left(x^{s}-1\right)$, $g\left(x\right)|\left(x^{t}-1\right),\left(x-1\right)∤f_{1}\left(x\right),\left(x-1\right)∤g\left(x\right)$.*
*2.*  *If $\lambda_{1}=\lambda_{2}=-1$, then $C=\langle \left(\left(x+1\right)f_{1}\left(x\right),0\right),\left(f_{1}\left(x\right),g\left(x\right)\right)\rangle$, where $f_{1}\left(x\right)|\left(x^{s}+1\right)$, $g\left(x\right)|\left(x^{t}+1\right),\left(x+1\right)∤f_{1}\left(x\right),\left(x+1\right)∤g\left(x\right)$.*

**Lemma** **7.** *Let C be a non-separable $F_{q}R$-$\left(\lambda_{1},\lambda_{2}\right)$-constacyclic code of block length (s,t).*

*1.*  *If $C=\langle \left(\left(x-1\right)f_{1}\left(x\right),0\right),\left(f_{1}\left(x\right),g\left(x\right)\right)\rangle$, then*
$$C^{⊥}=\langle \left(\frac{x^{s}-1}{f_{1}^{*}\left(x\right)},0\right),\left(-\frac{x^{s}-1}{\left(x-1\right)f_{1}^{*}\left(x\right)},e_{1}\frac{x^{t}-1}{\left(x-1\right)g_{1}^{*}\left(x\right)}+\sum_{i=2}^{4}e_{i}{\left(\frac{x^{t}-1}{g_{i}\left(x\right)}\right)}^{*}\right)\rangle ,$$
  *where $f_{1}\left(x\right)|\left(x^{s}-1\right),g\left(x\right)|\left(x^{t}-1\right),\left(x-1\right)∤f_{1}\left(x\right),\left(x-1\right)∤g\left(x\right)$, $g\left(x\right)=\sum_{i=1}^{4}e_{i}g_{i}\left(x\right)$.*
*2.*  *If $C=\langle \left(\left(x+1\right)f_{1}\left(x\right),0\right),\left(f_{1}\left(x\right),g\left(x\right)\right)\rangle$, then when $\deg(f_{1}\left(x\right))$ and $\deg(g_{1}\left(x\right))$ have opposite parity,*
$$C^{⊥}=\langle \left(\frac{x^{s}+1}{f_{1}^{*}\left(x\right)},0\right),\left(-\frac{x^{s}+1}{\left(x+1\right)f_{1}^{*}\left(x\right)},e_{1}\frac{x^{t}+1}{\left(x+1\right)g_{1}^{*}\left(x\right)}+\sum_{i=2}^{4}e_{i}{\left(\frac{x^{t}+1}{g_{i}\left(x\right)}\right)}^{*}\right)\rangle ;$$
  *when $\deg(f_{1}\left(x\right))$ and $\deg(g_{1}\left(x\right))$ have the same parity,*
$$C^{⊥}=\langle \left(\frac{x^{s}+1}{f_{1}^{*}\left(x\right)},0\right),\left(\frac{x^{s}+1}{\left(x+1\right)f_{1}^{*}\left(x\right)},e_{1}\frac{x^{t}+1}{\left(x+1\right)g_{1}^{*}\left(x\right)}+\sum_{i=2}^{4}e_{i}{\left(\frac{x^{t}+1}{g_{i}\left(x\right)}\right)}^{*}\right)\rangle ,$$
  *where $f_{1}\left(x\right)|\left(x^{s}+1\right),g\left(x\right)|\left(x^{t}+1\right),\left(x+1\right)∤f_{1}\left(x\right),\left(x+1\right)∤g\left(x\right)$, $g\left(x\right)=\sum_{i=1}^{4}e_{i}g_{i}\left(x\right)$.*

**Proof.**  As the proof approaches for Case 1 and Case 2 are analogous, we focus on proving Case 2.

Set $\lambda_{1}=\lambda_{2}=-1$, $f\left(x\right)=\left(x+1\right)f_{1}\left(x\right)$ and $l\left(x\right)=f_{1}\left(x\right)$; then,

$$\tilde{f}\left(x\right)={\left(\frac{x^{s}-\lambda_{1}}{\gcd(f(x),l(x))}\right)}^{*}={\left(\frac{x^{s}+1}{\gcd(\left(x+1\right)f_{1}\left(x\right),f_{1}\left(x\right))}\right)}^{*}=\frac{x^{s}+1}{f_{1}^{*}\left(x\right)},$$

$$\begin{array}{cc} {\tilde{g}}_{1}\left(x\right) & =\frac{\left(x^{t}-\lambda_{2}\right)\gcd{\left(f\left(x\right),l\left(x\right)\right)}^{*}}{f^{*}\left(x\right)g_{1}^{*}\left(x\right)}=\frac{\left(x^{t}+1\right)\gcd{\left(\left(x+1\right)f_{1}\left(x\right),f_{1}\left(x\right)\right)}^{*}}{{\left(\left(x+1\right)f_{1}\left(x\right)\right)}^{*}g_{1}^{*}\left(x\right)}\\ \; & =\frac{\left(x^{t}+1\right)f_{1}^{*}\left(x\right)}{{\left(\left(x+1\right)f_{1}\left(x\right)\right)}^{*}g_{1}^{*}\left(x\right)}=\frac{x^{t}+1}{\left(x+1\right)g_{1}^{*}\left(x\right)}, \end{array}$$

and

$${\tilde{g}}_{i}\left(x\right)={\left(\frac{x^{t}-\lambda_{2}}{g_{i}\left(x\right)}\right)}^{*}={\left(\frac{x^{t}+1}{g_{i}\left(x\right)}\right)}^{*}$$

for i=2,3,4.

When $\deg(f_{1}\left(x\right))$ and $\deg(g_{1}\left(x\right))$ have opposite parity, then

$$-x^{\mathrm{lcm}\left(s,t\right)-\deg\left(g_{1}\left(x\right)\right)+\deg\left(f_{1}\left(x\right)\right)}+1\equiv 0\left(\mathrm{mod}\left(x+1\right)\right),$$

so

μ(x)=−1,

We have

$$\tilde{l}\left(x\right)=\frac{x^{s}-\lambda_{1}}{f^{*}\left(x\right)}\mu \left(x\right)=-\frac{x^{s}+1}{f^{*}\left(x\right)}=-\frac{x^{s}+1}{\left(x+1\right)f_{1}^{*}\left(x\right)}.$$

By Lemma 6, we can have

$$C=\langle \left(\left(x+1\right)f_{1}\left(x\right),0\right),\left(f_{1}\left(x\right),g\left(x\right)\right)\rangle .$$

Let $\tilde{g}\left(x\right)=\sum_{i=1}^{4}e_{i}{\tilde{g}}_{i}\left(x\right)$; by Theorem 6, we have

$$\begin{array}{cc} C^{⊥} & =\langle \left(\tilde{f}\left(x\right),0\right),\left(\tilde{l}\left(x\right),\tilde{g}\left(x\right)\right)\rangle \\ \; & =\langle \left(\frac{x^{s}+1}{f_{1}^{*}\left(x\right)},0\right),\left(-\frac{x^{s}+1}{\left(x+1\right)f_{1}^{*}\left(x\right)},e_{1}\frac{x^{t}+1}{\left(x+1\right)g_{1}^{*}\left(x\right)}+\sum_{i=2}^{4}e_{i}{\left(\frac{x^{t}+1}{g_{i}\left(x\right)}\right)}^{*}\right)\rangle . \end{array}$$

Similarly, when $\deg(f_{1}\left(x\right))$ and $\deg(g_{1}\left(x\right))$ have the same parity, then

$$-x^{\mathrm{lcm}\left(s,t\right)-\deg\left(g_{1}\left(x\right)\right)+\deg\left(f_{1}\left(x\right)\right)}-1\equiv 0\left(\mathrm{mod}\left(x+1\right)\right),$$

We have

μ(x)=1,

and hence

$$C^{⊥}=\langle \left(\frac{x^{s}+1}{f_{1}^{*}\left(x\right)},0\right),\left(\frac{x^{s}+1}{\left(x+1\right)f_{1}^{*}\left(x\right)},e_{1}\frac{x^{t}+1}{\left(x+1\right)g_{1}^{*}\left(x\right)}+\sum_{i=2}^{4}e_{i}{\left(\frac{x^{t}+1}{g_{i}\left(x\right)}\right)}^{*}\right)\rangle .$$

□

**Lemma** **8.** *If*

$$\left(p_{1}\left(x\right)\mathrm{lcm}\left(f_{1}\left(x\right),\tilde{f_{1}}\left(x\right)\right),q_{1}\left(x\right)\sum_{i=1}^{4}e_{i}\mathrm{lcm}\left(g_{i}\left(x\right),\tilde{g_{i}}\left(x\right)\right)\right)\circ \left(f_{1}\left(x\right),g\left(x\right)\right)\equiv 0\left(\mathrm{mod}\left(x^{l}+1\right)\right),$$

where $\tilde{f_{1}}\left(x\right)=\frac{x^{s}+1}{\left(x+1\right)f_{1}^{*}\left(x\right)}$, $\tilde{g_{1}}\left(x\right)=\frac{x^{t}+1}{\left(x+1\right)g_{1}^{*}\left(x\right)}$, $\tilde{g_{i}}\left(x\right)={\left(\frac{x^{t}+1}{g_{i}\left(x\right)}\right)}^{*}$ for i=2,3,4, then

*1.*  When $\left(f_{1}\left(x\right),f_{1}^{*}\left(x\right)\right)$ and $\left(g_{1}\left(x\right),g_{1}^{*}\left(x\right)\right)$ are reciprocal pairs, if $\deg(f_{1}\left(x\right))$ and $\deg(g_{1}\left(x\right))$ have opposite parity, then $p_{1}\left(x\right)-q_{1}\left(x\right)\equiv 0\left(\mathrm{mod}\left(x+1\right)\right)$; if $\deg(f_{1}\left(x\right))$ and $\deg(g_{1}\left(x\right))$ have the same parity, then $p_{1}\left(x\right)+q_{1}\left(x\right)\equiv 0\left(\mathrm{mod}\left(x+1\right)\right)$.
*2.*  When $\left(f_{1}\left(x\right),f_{1}^{*}\left(x\right)\right)$ is a reciprocal pair, $g_{1}\left(x\right)$ is a self-reciprocal polynomial; if $\deg(f_{1}\left(x\right))$ and $\deg(g_{1}\left(x\right))$ have opposite parity, then $p_{1}\left(x\right)-q_{1}\left(x\right)g_{1}\left(x\right)\equiv 0\left(\mathrm{mod}\left(x+1\right)\right)$; if $\deg(f_{1}\left(x\right))$ and $\deg(g_{1}\left(x\right))$ have the same parity, then $p_{1}\left(x\right)+q_{1}\left(x\right)g_{1}\left(x\right)\equiv 0\left(\mathrm{mod}\left(x+1\right)\right)$.
*3.*  When $\left(g_{1}\left(x\right),g_{1}^{*}\left(x\right)\right)$ is a reciprocal pair, $f_{1}\left(x\right)$ is a self-reciprocal polynomial; if $\deg(f_{1}\left(x\right))$ and $\deg(g_{1}\left(x\right))$ have opposite parity, then $f_{1}\left(x\right)p_{1}\left(x\right)-q_{1}\left(x\right)\equiv 0\left(\mathrm{mod}\left(x+1\right)\right)$; if $\deg(f_{1}\left(x\right))$ and $\deg(g_{1}\left(x\right))$ have the same parity, then $f_{1}\left(x\right)p_{1}\left(x\right)+q_{1}\left(x\right)\equiv 0\left(\mathrm{mod}\left(x+1\right)\right)$.
*4.*  *When $g_{1}\left(x\right)$ and $f_{1}\left(x\right)$ are self-reciprocal polynomials, if $\deg(f_{1}\left(x\right))$ and $\deg(g_{1}\left(x\right))$ have opposite parity, then $f_{1}\left(x\right)p_{1}\left(x\right)-q_{1}\left(x\right)g_{1}\left(x\right)\equiv 0\left(\mathrm{mod}\left(x+1\right)\right)$; if $\deg(f_{1}\left(x\right))$ and $\deg(g_{1}\left(x\right))$ have the same parity, then $f_{1}\left(x\right)p_{1}\left(x\right)+q_{1}\left(x\right)g_{1}\left(x\right)\equiv 0\left(\mathrm{mod}\left(x+1\right)\right)$.*

**Proof.**  As the proof approaches from Case 1 to Case 4 are analogous, we focus on proving Case 1.

If

$$\left(p_{1}\left(x\right)\mathrm{lcm}\left(f_{1}\left(x\right),\tilde{f_{1}}\left(x\right)\right),q_{1}\left(x\right)\sum_{i=1}^{4}e_{i}\mathrm{lcm}\left(g_{i}\left(x\right),\tilde{g_{i}}\left(x\right)\right)\right)\circ \left(f_{1}\left(x\right),g\left(x\right)\right)\equiv 0\left(\mathrm{mod}\left(x^{l}+1\right)\right),$$

then

$$\begin{array}{cc} \; & e_{1}p_{1}\left(x\right)\mathrm{lcm}\left(f_{1}\left(x\right),\tilde{f_{1}}\left(x\right)\right)f_{1}^{*}\left(x\right)x^{l-\deg(f_{1}\left(x\right))-1}\frac{x^{l}+1}{x^{s}+1}+\\ \; & q_{1}\left(x\right)\sum_{i=1}^{4}e_{i}\mathrm{lcm}\left(g_{i}\left(x\right),\tilde{g_{i}}\left(x\right)\right)g^{*}\left(x\right)x^{l-\deg(g(x))-1}\frac{x^{l}+1}{x^{t}+1}\\ \; & \equiv 0(\mathrm{mod}\left(x^{l}+1\right)), \end{array}$$

which implies

$$\begin{array}{cc} \; & e_{1}p_{1}\left(x\right)\mathrm{lcm}\left(f_{1}\left(x\right),\frac{x^{s}+1}{\left(x+1\right)f_{1}^{*}\left(x\right)}\right)f_{1}^{*}\left(x\right)x^{l-\deg(f_{1}\left(x\right))-1}\frac{x^{l}+1}{x^{s}+1}+\\ \; & e_{1}q_{1}\left(x\right)\mathrm{lcm}\left(g_{1}\left(x\right),\frac{x^{t}+1}{\left(x+1\right)g_{1}^{*}\left(x\right)}\right)x^{\deg\left(g\left(x\right)\right)-\deg\left(g_{1}\left(x\right)\right)}g_{1}^{*}\left(x\right)x^{l-\deg(g(x))-1}\frac{x^{l}+1}{x^{t}+1}\\ \; & \equiv 0(\mathrm{mod}\left(x^{l}+1\right)), \end{array}$$

Hence,

$$\begin{array}{cc} \; & p_{1}\left(x\right)\mathrm{lcm}\left(f_{1}\left(x\right),\frac{x^{s}+1}{\left(x+1\right)f_{1}^{*}\left(x\right)}\right)f_{1}^{*}\left(x\right)x^{l-\deg(f_{1}\left(x\right))-1}\frac{x^{l}+1}{x^{s}+1}+\\ \; & q_{1}\left(x\right)\mathrm{lcm}\left(g_{1}\left(x\right),\frac{x^{t}+1}{\left(x+1\right)g_{1}^{*}\left(x\right)})\right)g_{1}^{*}\left(x\right)x^{l-\deg(g_{1}\left(x\right))-1}\frac{x^{l}+1}{x^{t}+1}\\ \; & \equiv 0(\mathrm{mod}\left(x^{l}+1\right)), \end{array}$$

Therefore,

$$\begin{array}{cc} \; & p_{1}\left(x\right)x^{l-\deg(f_{1}\left(x\right))-1}\frac{x^{l}+1}{x+1}+q_{1}\left(x\right)x^{l-\deg(g_{1}\left(x\right))-1}\frac{x^{l}+1}{x+1}\\ \; & =\frac{x^{l}+1}{x+1}\left(p_{1}\left(x\right)x^{l-\deg(f_{1}\left(x\right))-1}+q_{1}\left(x\right)x^{l-\deg(g_{1}\left(x\right))-1}\right)\\ \; & \equiv 0(\mathrm{mod}\left(x^{l}+1\right)). \end{array}$$

Since $\left(x+1\right)|\left(x^{l}+1\right)$,

$$p_{1}\left(x\right)x^{l-\deg(f_{1}\left(x\right))-1}+q_{1}\left(x\right)x^{l-\deg(g_{1}\left(x\right))-1}\equiv 0\left(\mathrm{mod}\left(x+1\right)\right).$$

If $\deg(f_{1}\left(x\right))$ and $\deg(g_{1}\left(x\right))$ have opposite parity, for example, if $\deg(f_{1}\left(x\right))$ is even and $\deg(g_{1}\left(x\right))$ is odd, then

$$x^{l-\deg(f_{1}\left(x\right))-1}\equiv 1\left(\mathrm{mod}\left(x+1\right)\right)$$

and

$$x^{l-\deg(g_{1}\left(x\right))-1}\equiv -1\left(\mathrm{mod}\left(x+1\right)\right),$$

so

$$p_{1}\left(x\right)-q_{1}\left(x\right)\equiv 0\left(\mathrm{mod}\left(x+1\right)\right).$$

Similarly, if $\deg(f_{1}\left(x\right))$ and $\deg(g_{1}\left(x\right))$ have the same parity, for example, if $\deg(f_{1}\left(x\right))$ and $\deg(g_{1}\left(x\right))$ are odd, then

$$x^{l-\deg(f_{1}\left(x\right))-1}\equiv -1\left(\mathrm{mod}\left(x+1\right)\right)$$

and

$$x^{l-\deg(g_{1}\left(x\right))-1}\equiv -1\left(\mathrm{mod}\left(x+1\right)\right),$$

so

$$p_{1}\left(x\right)+q_{1}\left(x\right)\equiv 0\left(\mathrm{mod}\left(x+1\right)\right).$$

□

Using the same method as in Lemma 8, we can obtain the following Lemma.

**Lemma** **9.**
$$\left(p_{1}\left(x\right)\mathrm{lcm}\left(f_{1}\left(x\right),\tilde{f_{1}}\left(x\right)\right),q_{1}\left(x\right)\sum_{i=1}^{4}e_{i}\mathrm{lcm}\left(g_{i}\left(x\right),\tilde{g_{i}}\left(x\right)\right)\right)\circ \left(f_{1}\left(x\right),g\left(x\right)\right)\equiv 0\left(\mathrm{mod}\left(x^{l}-1\right)\right),$$

where $g\left(x\right)=\sum_{i=1}^{4}e_{i}g_{i}\left(x\right)$, $\tilde{f_{1}}\left(x\right)=\frac{x^{s}-1}{\left(x-1\right)f_{1}^{*}\left(x\right)}$, $\tilde{g_{1}}\left(x\right)=\frac{x^{t}-1}{\left(x-1\right)g_{1}^{*}\left(x\right)}$, $\tilde{g_{i}}\left(x\right)={\left(\frac{x^{t}-1}{g_{i}\left(x\right)}\right)}^{*}$ for i=2,3,4.

*1.*  When $\left(f_{1}\left(x\right),f_{1}^{*}\left(x\right)\right)$ and $\left(g_{1}\left(x\right),g_{1}^{*}\left(x\right)\right)$ are reciprocal pairs, then $p_{1}\left(x\right)+q_{1}\left(x\right)\equiv 0\left(\mathrm{mod}\left(x-1\right)\right)$.
*2.*  When $\left(f_{1}\left(x\right),f_{1}^{*}\left(x\right)\right)$ is a reciprocal pair, $g_{1}\left(x\right)$ is a self-reciprocal polynomial, and $p_{1}\left(x\right)+q_{1}\left(x\right)g_{1}\left(x\right)\equiv 0\left(\mathrm{mod}\left(x-1\right)\right)$.
*3.*  When $\left(g_{1}\left(x\right),g_{1}^{*}\left(x\right)\right)$ is a reciprocal pair, $f_{1}\left(x\right)$ is a self-reciprocal polynomial, and $f_{1}\left(x\right)p_{1}\left(x\right)+q_{1}\left(x\right)\equiv 0\left(\mathrm{mod}\left(x-1\right)\right)$.
*4.*  *When $g_{1}\left(x\right)$ and $f_{1}\left(x\right)$ are self-reciprocal polynomials, then $f_{1}\left(x\right)p_{1}\left(x\right)+q_{1}\left(x\right)g_{1}\left(x\right)\equiv 0\left(\mathrm{mod}\left(x-1\right)\right)$.*

**Theorem** **7.** *Let C be a non-separable $F_{q}R$-$\left(\lambda_{1},\lambda_{2}\right)$-constacyclic code of block length (s,t).*

*1.*  *If $C=\langle \left(\left(x-1\right)f_{1}\left(x\right),0\right),\left(f_{1}\left(x\right),g\left(x\right)\right)\rangle$, then*
$$\mathrm{Hull}\left(C\right)=\langle \left(\mathrm{lcm}\left(\left(x-1\right)f_{1}\left(x\right),\frac{x^{s}-1}{f_{1}^{*}\left(x\right)}\right),0\right),\left(-\mathrm{lcm}\left(f_{1}\left(x\right),\tilde{f_{1}}\left(x\right)\right),\sum_{i=1}^{4}e_{i}\mathrm{lcm}\left(g_{i}\left(x\right),\tilde{g_{i}}\left(x\right)\right)\right)\rangle ,$$
  where $f_{1}\left(x\right)|\left(x^{s}-1\right),g\left(x\right)|\left(x^{t}-1\right),\left(x-1\right)∤f_{1}\left(x\right),\left(x-1\right)∤g\left(x\right)$, $g\left(x\right)=\sum_{i=1}^{4}e_{i}g_{i}\left(x\right)$, $\tilde{f_{1}}\left(x\right)=\frac{x^{s}-1}{\left(x-1\right)f_{1}^{*}\left(x\right)}$, $\tilde{g_{1}}\left(x\right)=\frac{x^{t}-1}{\left(x-1\right)g_{1}^{*}\left(x\right)}$, $\tilde{g_{i}}\left(x\right)={\left(\frac{x^{t}-1}{g_{i}\left(x\right)}\right)}^{*}$ for i=2,3,4.
*2.*  *If $C=\langle \left(\left(x+1\right)f_{1}\left(x\right),0\right),\left(f_{1}\left(x\right),g\left(x\right)\right)\rangle$, then when $\deg(f_{1}\left(x\right))$ and $\deg(g_{1}\left(x\right))$ have the same parity,*
$$\mathrm{Hull}\left(C\right)=\langle \left(\mathrm{lcm}\left(\left(x+1\right)f_{1}\left(x\right),\frac{x^{s}+1}{f_{1}^{*}\left(x\right)}\right),0\right),\left(-\mathrm{lcm}\left(f_{1}\left(x\right),\tilde{f_{1}}\left(x\right)\right),\sum_{i=1}^{4}e_{i}\mathrm{lcm}\left(g_{i}\left(x\right),\tilde{g_{i}}\left(x\right)\right)\right)\rangle ;$$
  *when $\deg(f_{1}\left(x\right))$ and $\deg(g_{1}\left(x\right))$ have opposite parity,*
$$\mathrm{Hull}\left(C\right)=\langle \left(\mathrm{lcm}\left(\left(x+1\right)f_{1}\left(x\right),\frac{x^{s}+1}{f_{1}^{*}\left(x\right)}\right),0\right),\left(\mathrm{lcm}\left(f_{1}\left(x\right),\tilde{f_{1}}\left(x\right)\right),\sum_{i=1}^{4}e_{i}\mathrm{lcm}\left(g_{i}\left(x\right),\tilde{g_{i}}\left(x\right)\right)\right)\rangle ,$$
  where $f_{1}\left(x\right)|\left(x^{s}+1\right),g\left(x\right)|\left(x^{t}+1\right),\left(x+1\right)∤f_{1}\left(x\right),\left(x+1\right)∤g\left(x\right)$, $g\left(x\right)=\sum_{i=1}^{4}e_{i}g_{i}\left(x\right)$, $\tilde{f_{1}}\left(x\right)=\frac{x^{s}+1}{\left(x+1\right)f_{1}^{*}\left(x\right)}$, $\tilde{g_{1}}\left(x\right)=\frac{x^{t}+1}{\left(x+1\right)g_{1}^{*}\left(x\right)}$, $\tilde{g_{i}}\left(x\right)={\left(\frac{x^{t}+1}{g_{i}\left(x\right)}\right)}^{*}$ for i=2,3,4.

**Proof.**  As the proof approaches for Case 1 and Case 2 are analogous, we focus on proving Case 2.

If $\left(f_{1}\left(x\right),f_{1}^{*}\left(x\right)\right)$ and $\left(g_{1}\left(x\right),g_{1}^{*}\left(x\right)\right)$ are reciprocal pairs, $\deg(f_{1}\left(x\right))$ and $\deg(g_{1}\left(x\right))$ have the same parity, and Hull(C) is a non-separable constacyclic code of block length (s,t).

By Lemma 6, we suppose

$$\mathrm{Hull}\left(C\right)=\langle \left((x+1)a(x),0\right),\left(a(x),b(x)\right)\rangle ,$$

where $a\left(x\right)|\left(x^{s}+1\right),b\left(x\right)|\left(x^{t}+1\right),\left(x+1\right)∤a\left(x\right),\left(x+1\right)∤b\left(x\right)$.

Furthermore, by Lemma 3 and Lemma 5,

$${\left(\mathrm{Hull}\left(C\right)\right)}_{s}=\langle a\left(x\right)\rangle \subseteq \langle f_{1}\left(x\right)\rangle ⋂\langle \tilde{f_{1}}\left(x\right)\rangle =\langle \mathrm{lcm}\left(f_{1}\left(x\right),\tilde{f_{1}}\left(x\right)\right)\rangle ,$$

and

$${\left(\mathrm{Hull}\left(C\right)\right)}_{t}=\langle b\left(x\right)\rangle \subseteq \langle \sum_{i=1}^{4}e_{i}g_{i}\left(x\right)\rangle \;⋂\;\langle \sum_{i=1}^{4}e_{i}\tilde{g_{i}}\left(x\right)\rangle =\langle \sum_{i=1}^{4}e_{i}\mathrm{lcm}\left(g_{i}\left(x\right),\tilde{g_{i}}\left(x\right)\right)\rangle ,$$

It follows that

$$\mathrm{lcm}\left(f_{1}\left(x\right),\tilde{f_{1}}\left(x\right)\right)|a\left(x\right)$$

and

$$\sum_{i=1}^{4}e_{i}\mathrm{lcm}\left(g_{i}\left(x\right),\tilde{g_{i}}\left(x\right)\right)|b\left(x\right).$$

Let

$$a\left(x\right)=p_{1}\left(x\right)\mathrm{lcm}\left(f_{1}\left(x\right),\tilde{f_{1}}\left(x\right)\right)$$

and

$$b\left(x\right)=q_{1}\left(x\right)\sum_{i=1}^{4}e_{i}\mathrm{lcm}\left(g_{i}\left(x\right),\tilde{g_{i}}\left(x\right)\right),$$

where $p_{1}\left(x\right),q_{1}\left(x\right)\in F_{q}\left[x\right]$.

Therefore,

$$\begin{array}{cc} \; & \mathrm{Hull}(C)=\\ \; & \langle \left(p_{1}\left(x\right)\mathrm{lcm}\left(\left(x+1\right)f_{1}\left(x\right),\frac{x^{s}+1}{f_{1}^{*}\left(x\right)}\right),0\right),\left(p_{1}\left(x\right)\mathrm{lcm}\left(f_{1}\left(x\right),\tilde{f_{1}}\left(x\right)\right),q_{1}\left(x\right)\sum_{i=1}^{4}e_{i}\mathrm{lcm}\left(g_{i}\left(x\right),\tilde{g_{i}}\left(x\right)\right)\right)\rangle . \end{array}$$

Let

$$\tilde{C}=\langle \left(a\mathrm{lcm}\left(\left(x+1\right)f_{1}\left(x\right),\frac{x^{s}+1}{f_{1}^{*}\left(x\right)}\right),0\right),\left(a\mathrm{lcm}\left(f_{1}\left(x\right),\tilde{f_{1}}\left(x\right)\right),b\sum_{i=1}^{4}e_{i}\mathrm{lcm}\left(g_{i}\left(x\right),\tilde{g_{i}}\left(x\right)\right)\right)\rangle ,$$

where $a\equiv p_{1}\left(x\right)\left(\mathrm{mod}\left(x+1\right)\right)$, $b\equiv q_{1}\left(x\right)\left(\mathrm{mod}\left(x+1\right)\right),a,b\in F_{q}$.

In the following, we will prove that

$$\mathrm{Hull}\left(C\right)=\tilde{C}.$$

Since

$$\left(p_{1}\left(x\right)\mathrm{lcm}\left(f_{1}\left(x\right),\tilde{f_{1}}\left(x\right)\right),q_{1}\left(x\right)\sum_{i=1}^{4}e_{i}\mathrm{lcm}\left(g_{i}\left(x\right),\tilde{g_{i}}\left(x\right)\right)\right)\circ \left(f_{1}\left(x\right),g\left(x\right)\right)\equiv 0\left(\mathrm{mod}\left(x^{l}+1\right)\right),$$

by Lemma 8, we have

$$p_{1}\left(x\right)+q_{1}\left(x\right)\equiv 0\left(\mathrm{mod}\left(x+1\right)\right).$$

Let

$$p_{1}\left(x\right)+q_{1}\left(x\right)=a_{1}\left(x\right)\left(x+1\right),$$

$$p_{1}\left(x\right)=a+\alpha \left(x\right)\left(x+1\right)$$

and

$$q_{1}\left(x\right)=b+\beta \left(x\right)\left(x+1\right),$$

Then,

a+b≡0(mod(x+1)),

We have

a=−b≠0,

so

$$\begin{array}{cc} \; & \left(p_{1}\left(x\right)\mathrm{lcm}\left(f_{1}\left(x\right),\tilde{f_{1}}\left(x\right)\right),q_{1}\left(x\right)\sum_{i=1}^{4}e_{i}\mathrm{lcm}\left(g_{i}\left(x\right),\tilde{g_{i}}\left(x\right)\right)\right)\\ \; & =q_{1}\left(x\right)b^{-1}★\left(a\mathrm{lcm}\left(f_{1}\left(x\right),\tilde{f_{1}}\left(x\right)\right),b\sum_{i=1}^{4}e_{i}\mathrm{lcm}\left(g_{i}\left(x\right),\tilde{g_{i}}\left(x\right)\right)\right)\\ \; & \;\;\;\;+a_{1}\left(x\right)a^{-1}★\left(a\mathrm{lcm}\left(\left(x+1\right)f_{1}\left(x\right),\tilde{f_{1}}\left(x\right)\right),0\right)\\ \; & \in \tilde{C}. \end{array}$$

And since

$$\left(p_{1}\left(x\right)\mathrm{lcm}\left(\left(x+1\right)f_{1}\left(x\right),\frac{x^{s}+1}{f_{1}^{*}\left(x\right)}\right),0\right)\in \tilde{C},$$

hence,

$$\mathrm{Hull}\left(C\right)\subseteq \tilde{C}.$$

Conversely, since

$$\left(\tilde{f_{1}}\left(x\right),\sum_{i=1}^{4}e_{i}\tilde{g_{i}}\left(x\right)\right)\in C^{⊥}$$

and

$$\left(a\mathrm{lcm}\left(f_{1}\left(x\right),\tilde{f_{1}}\left(x\right)\right),b\sum_{i=1}^{4}e_{i}\mathrm{lcm}\left(g_{i}\left(x\right),\tilde{g_{i}}\left(x\right)\right)\right)\in \tilde{C},$$

we can get

$$\begin{array}{cc} \; & \left(alcm\left(f_{1}\left(x\right),\tilde{f_{1}}\left(x\right)\right),b\sum_{i=1}^{4}e_{i}\mathrm{lcm}\left(g_{i}\left(x\right),\tilde{g_{i}}\left(x\right)\right)\right)\circ \left(\tilde{f_{1}}\left(x\right),\sum_{i=1}^{4}e_{i}\tilde{g_{i}}\left(x\right)\right)\\ \; & \equiv e_{1}\frac{x^{l}+1}{x+1}\left(ax^{l-1-\deg(\tilde{f_{1}}\left(x\right))}+bx^{l-1-\deg(\tilde{g_{1}}\left(x\right))}\right)\left(\mathrm{mod}\left(x^{l}+1\right)\right). \end{array}$$

Since $\deg(f_{1}\left(x\right))$ and $\deg(g_{1}\left(x\right))$ have the same parity, $\deg(\tilde{f_{1}}\left(x\right))$ and $\deg(\tilde{g_{1}}\left(x\right))$ have the same parity; for example, if $\deg(\tilde{f_{1}}\left(x\right))$ and $\deg(\tilde{g_{1}}\left(x\right))$ are odd, then

$$x^{l-\deg(\tilde{f_{1}}\left(x\right))-1}\equiv -1\left(\mathrm{mod}\left(x+1\right)\right)$$

and

$$x^{l-\deg(\tilde{g_{1}}\left(x\right))-1}\equiv -1\left(\mathrm{mod}\left(x+1\right)\right),$$

so

$$\frac{x^{l}+1}{x+1}\left(ax^{l-1-\deg(\tilde{f_{1}}\left(x\right))}+bx^{l-1-\deg(\tilde{g_{1}}\left(x\right))}\right)\equiv 0\left(\mathrm{mod}\left(x^{l}+1\right)\right),$$

We have

$$\left(a\mathrm{lcm}\left(f_{1}\left(x\right),\tilde{f_{1}}\left(x\right)\right),b\sum_{i=1}^{4}e_{i}\mathrm{lcm}\left(g_{i}\left(x\right),\tilde{g_{i}}\left(x\right)\right)\right)\circ \left(\tilde{f_{1}}\left(x\right),\sum_{i=1}^{4}e_{i}\tilde{g_{i}}\left(x\right)\right)\equiv 0\left(\mathrm{mod}\left(x^{l}+1\right)\right).$$

so

$$\left(a\mathrm{lcm}\left(f_{1}\left(x\right),\tilde{f_{1}}\left(x\right)\right),b\sum_{i=1}^{4}e_{i}\mathrm{lcm}\left(g_{i}\left(x\right),\tilde{g_{i}}\left(x\right)\right)\right)\in C.$$

It is evident that

$$\left(\mathrm{lcm}\left(\left(x+1\right)f_{1}\left(x\right),\frac{x^{s}+1}{f_{1}^{*}\left(x\right)}\right),0\right)\in C,$$

so

$$\tilde{C}\subseteq C.$$

Since

$$(f_{1}\left(x\right),g\left(x\right))\in C,$$

and

$$\left(alcm\left(f_{1}\left(x\right),\tilde{f_{1}}\left(x\right)\right),b\sum_{i=1}^{4}e_{i}\mathrm{lcm}\left(g_{i}\left(x\right),\tilde{g_{i}}\left(x\right)\right)\right)\in \tilde{C},$$

we can get

$$\begin{array}{cc} \; & \left(alcm\left(f_{1}\left(x\right),\tilde{f_{1}}\left(x\right)\right),b\sum_{i=1}^{4}e_{i}\mathrm{lcm}\left(g_{i}\left(x\right),\tilde{g_{i}}\left(x\right)\right)\right)\circ \left(f_{1}\left(x\right),g\left(x\right)\right)\\ \; & \equiv e_{1}\frac{x^{l}+1}{x+1}\left(ax^{l-\deg(f_{1}\left(x\right))-1}+bx^{l-\deg(g_{1}\left(x\right))-1}\right)\left(\mathrm{mod}\left(x^{l}+1\right)\right). \end{array}$$

Since $\deg(f_{1}\left(x\right))$ and $\deg(g_{1}\left(x\right))$ have the same parity, for example, $\deg(f_{1}\left(x\right))$ and $\deg(g_{1}\left(x\right))$ are even, then

$$x^{l-\deg(f_{1}\left(x\right))-1}\equiv 1\left(\mathrm{mod}\left(x+1\right)\right)$$

and

$$x^{l-\deg(g_{1}\left(x\right))-1}\equiv 1\left(\mathrm{mod}\left(x+1\right)\right),$$

so

$$\frac{x^{l}+1}{x+1}\left(ax^{l-\deg(f_{1}\left(x\right))-1}+bx^{l-\deg(g_{1}\left(x\right))-1}\right)\equiv 0\left(\mathrm{mod}\left(x^{l}+1\right)\right).$$

We have

$$\left(alcm\left(f_{1}\left(x\right),\tilde{f_{1}}\left(x\right)\right),b\sum_{i=1}^{4}e_{i}\mathrm{lcm}\left(g_{i}\left(x\right),\tilde{g_{i}}\left(x\right)\right)\right)\circ \left(f_{1}\left(x\right),g\left(x\right)\right)\equiv 0\left(\mathrm{mod}\left(x^{l}+1\right)\right),$$

and hence

$$\tilde{C}\subseteq C^{⊥},$$

that is,

$$\tilde{C}\subseteq \mathrm{Hull}\left(C\right).$$

So,

$$\tilde{C}=\mathrm{Hull}\left(C\right)$$

Furthermore, because

a=−b≠0,

hence,

$$\mathrm{Hull}\left(C\right)=\langle \left(\mathrm{lcm}\left(\left(x+1\right)f_{1}\left(x\right),\frac{x^{s}+1}{f_{1}^{*}\left(x\right)}\right),0\right),\left(-\mathrm{lcm}\left(f_{1}\left(x\right),\tilde{f_{1}}\left(x\right)\right),\sum_{i=1}^{4}e_{i}\mathrm{lcm}\left(g_{i}\left(x\right),\tilde{g_{i}}\left(x\right)\right)\right)\rangle .$$

□

By Theorem 5 and Theorem 7, we can have the following Corollary.

**Corollary** **2.** *Let C be a non-separable $F_{q}R$-$\left(\lambda_{1},\lambda_{2}\right)$-constacyclic code of block length (s,t).*

*1.*  *If $C=\langle \left(\left(x+1\right)f_{1}\left(x\right),0\right),\left(f_{1}\left(x\right),g\left(x\right)\right)\rangle$, then*
$$\dim\left(\mathrm{Hull}\left(C\right)\right)=s-\deg\left(\mathrm{lcm}\left(\left(x-1\right)f_{1}\left(x\right),\frac{x^{s}-1}{f_{1}^{*}\left(x\right)}\right)\right)+\sum_{i=1}^{4}\left(t-\deg\left(\mathrm{lcm}\left(g_{i}\left(x\right),\tilde{g_{i}}\left(x\right)\right)\right)\right),$$
  where $f_{1}\left(x\right)|\left(x^{s}-1\right),g\left(x\right)|\left(x^{t}-1\right),\left(x-1\right)∤f_{1}\left(x\right),\left(x-1\right)∤g\left(x\right)$, $g\left(x\right)=\sum_{i=1}^{4}e_{i}g_{i}\left(x\right)$, $\tilde{f_{1}}\left(x\right)=\frac{x^{s}-1}{\left(x-1\right)f_{1}^{*}\left(x\right)}$, $\tilde{g_{1}}\left(x\right)=\frac{x^{t}-1}{\left(x-1\right)g_{1}^{*}\left(x\right)}$, $\tilde{g_{i}}\left(x\right)={\left(\frac{x^{t}-1}{g_{i}\left(x\right)}\right)}^{*}$ for i=2,3,4.
*2.*  *If $C=\langle \left(\left(x+1\right)f_{1}\left(x\right),0\right),\left(f_{1}\left(x\right),g\left(x\right)\right)\rangle$, then*
$$\dim\left(\mathrm{Hull}\left(C\right)\right)=s-\deg\left(\mathrm{lcm}\left(\left(x+1\right)f_{1}\left(x\right),\frac{x^{s}+1}{f_{1}^{*}\left(x\right)}\right)\right)+\sum_{i=1}^{4}\left(t-\deg\left(\mathrm{lcm}\left(g_{i}\left(x\right),\tilde{g_{i}}\left(x\right)\right)\right)\right),$$
  where $f_{1}\left(x\right)|\left(x^{s}+1\right),g\left(x\right)|\left(x^{t}+1\right),\left(x+1\right)∤f_{1}\left(x\right),\left(x+1\right)∤g\left(x\right)$, $g\left(x\right)=\sum_{i=1}^{4}e_{i}g_{i}\left(x\right)$, $\tilde{f_{1}}\left(x\right)=\frac{x^{s}+1}{\left(x+1\right)f_{1}^{*}\left(x\right)}$, $\tilde{g_{1}}\left(x\right)=\frac{x^{t}+1}{\left(x+1\right)g_{1}^{*}\left(x\right)}$, $\tilde{g_{i}}\left(x\right)={\left(\frac{x^{t}+1}{g_{i}\left(x\right)}\right)}^{*}$ for i=2,3,4.

## 5. New QECCs

In this section, we construct some new QECCs using the quantum construction X method from the non-separable constacyclic codes over $F_{q}R$.

**Theorem** **8.** *Let C be an $F_{q}R$-$\left(\lambda_{1},\lambda_{2}\right)$-constacyclic code with parameters [s+t,k,d]. Then, ψ(C) is a linear code with parameters [s+4t,k,d], and $\psi {\left(C\right)}^{⊥}=\psi \left(C^{⊥}\right)$.*

**Proof.**  Based on Lemma 2 and Theorem 5, ψ(C) is a linear code with parameters [s+4t,k,d].

For any $a=(c_{0},c_{1},\cdots ,c_{s-1},d_{0},d_{1},\cdots ,d_{t-1})\in C$, $\tilde{a}=\left(c_{0}^{\prime },c_{1}^{\prime },\cdots ,c_{s-1}^{\prime },d_{0}^{\prime },d_{1}^{\prime },\cdots ,d_{t-1}^{\prime }\right)\in C^{⊥}$, where $d_{i}=d_{1,i}e_{1}+d_{2,i}e_{2}+d_{3,i}e_{3}+d_{4,i}e_{4}$, $d_{i}^{\prime }=d_{1,i}^{\prime }e_{1}+d_{2,i}^{\prime }e_{2}+d_{3,i}^{\prime }e_{3}+d_{4,i}^{\prime }e_{4}$, i=0,1,⋯,t−1. Let $c=(c_{0},c_{1},\cdots ,c_{s-1})$, $d^{\left(i\right)}=\left(d_{i,0},d_{i,1},\cdots ,d_{i,t-1}\right)$, $c^{\prime }=\left(c_{0}^{\prime },c_{1}^{\prime },\cdots ,c_{s-1}^{\prime }\right)$, $d^{\prime }\left(i\right)=\left(d_{i,0}^{\prime },d_{i,1}^{\prime },\cdots ,d_{i,t-1}^{\prime }\right)$; for i=1,2,3,4, we have

$$\begin{array}{c} \begin{array}{cc} 0 & =a\cdot \tilde{a}\\ & =e_{1}\sum_{i=0}^{s-1}c_{i}c_{i}^{\prime }+\sum_{j=0}^{t-1}d_{j}d_{j}^{\prime }\\ & =e_{1}\sum_{i=0}^{s-1}c_{i}c_{i}^{\prime }+e_{1}\sum_{j=0}^{t-1}d_{1,j}d_{1,j}^{\prime }+e_{2}\sum_{j=0}^{t-1}d_{2,j}d_{2,j}^{\prime }+e_{3}\sum_{j=0}^{t-1}d_{3,j}d_{3,j}^{\prime }+e_{4}\sum_{j=0}^{t-1}d_{4,j}d_{4,j}^{\prime }, \end{array} \end{array}$$

which implies

$$\sum_{i=0}^{s-1}c_{i}c_{i}^{\prime }+\sum_{j=0}^{t-1}d_{1,j}d_{1,j}^{\prime }=0$$

and

$$\sum_{j=0}^{t-1}d_{i,j}d_{i,j}^{\prime }=0$$

for i=2,3,4.

Thus,

$$\begin{array}{c} \begin{array}{cc} \psi \left(a\right)\cdot \psi \left(\tilde{a}\right) & =c\cdot c^{\prime }+d^{\left(1\right)}\cdot d^{\prime }\left(1\right)+d^{\left(2\right)}\cdot d^{\prime }\left(2\right)+d^{\left(3\right)}\cdot d^{\prime }\left(3\right)+d^{\left(4\right)}\cdot d^{\prime }\left(4\right)\\ & =\sum_{i=0}^{s-1}c_{i}c_{i}^{\prime }+\sum_{j=0}^{t-1}d_{1,j}d_{1,j}^{\prime }+\sum_{j=0}^{t-1}d_{2,j}d_{2,j}^{\prime }+\sum_{j=0}^{t-1}d_{3,j}d_{3,j}^{\prime }+\sum_{j=0}^{t-1}d_{4,j}d_{4,j}^{\prime }\\ & =0. \end{array} \end{array}$$

which implies

$$\psi \left(C^{⊥}\right)\subseteq \psi {\left(C\right)}^{⊥}.$$

Based on Lemma 2, ψ is a distance-preserving linear bijection from $F_{q}^{s}\times R^{t}$ to $F_{q}^{s+4t}$, so

$$|\psi \left(C^{⊥}\right)|=|C^{⊥}|=\frac{q^{s+4t}}{|C|}=\frac{q^{s+4t}}{|\psi (C)|}=|\psi {\left(C\right)}^{⊥}|,$$

Hence,

$$\psi {\left(C\right)}^{⊥}=\psi \left(C^{⊥}\right).$$

□

**Corollary** **3.** *Let C be an $F_{q}R$-$\left(\lambda_{1},\lambda_{2}\right)$-constacyclic code with parameters [s+t,k,d]. Then, ψ(Hull(C))=Hull(ψ(C)).*

**Proof.**  Based on Theorem 8, we have

$$\begin{array}{c} \psi {\left(C\right)}^{⊥}=\psi \left(C^{⊥}\right), \end{array}$$

so

$$\begin{array}{c} \mathrm{Hull}\left(\psi \left(C\right)\right)=\psi \left(C\right)\cap \psi \left(C^{⊥}\right). \end{array}$$

Next, we will prove that

$$\psi \left(\mathrm{Hull}\left(C\right)\right)=\psi \left(C\right)\cap \psi \left(C^{⊥}\right).$$

For any

$$a\in \psi \left(C\right)\cap \psi \left(C^{⊥}\right),$$

there exist c∈C and $d\in C^{⊥}$ such that

a=ψ(c)=ψ(d).

Based on Lemma 2, ψ is a bijection, which implies

$$c=d\in C\cap C^{⊥}.$$

Thus,

$$\begin{array}{c} a=\psi \left(c\right)\in \psi \left(C\cap C^{⊥}\right)=\psi \left(\mathrm{Hull}\left(C\right)\right), \end{array}$$

which implies

$$\begin{array}{c} \psi \left(C\right)\cap \psi \left(C^{⊥}\right)\subseteq \psi \left(\mathrm{Hull}\left(C\right)\right). \end{array}$$

Conversely, for any

$$a\in \psi \left(\mathrm{Hull}\left(C\right)\right)=\psi \left(C\cap C^{⊥}\right),$$

there exist $b\in C\cap C^{⊥}$ such that

a=ψ(b)∈ψ(C)

and

$$a=\psi \left(b\right)\in \psi \left(C^{⊥}\right).$$

Thus,

$$\begin{array}{c} a\in \psi \left(C\right)\cap \psi \left(C^{⊥}\right), \end{array}$$

and we can have

$$\begin{array}{c} \psi \left(\mathrm{Hull}\left(C\right)\right)\subseteq \psi \left(C\right)\cap \psi \left(C^{⊥}\right). \end{array}$$

Therefore,

$$\begin{array}{c} \psi \left(\mathrm{Hull}\left(C\right)\right)=\psi \left(C\right)\cap \psi \left(C^{⊥}\right)=\mathrm{Hull}\left(\psi \left(C\right)\right). \end{array}$$

□

**Lemma** **10.** *Let C be an $F_{q}R$-$\left(\lambda_{1},\lambda_{2}\right)$-constacyclic code of block length (s,t). Then, ${\left(C+C^{⊥}\right)}^{⊥}=\mathrm{Hull}\left(C\right)$.*

**Proof.**  For any $\;r\in {\left(C+C^{⊥}\right)}^{⊥}$ and any $\;a\in C,\;b\in C^{⊥}$, we have

r·(a+b)=0.

Let a=0; we have

r·b=0

which implies

r∈C;

let b=0; we have

r·a=0

which implies

$$r\in C^{⊥}.$$

Thus,

$$r\in C\cap C^{⊥}=\mathrm{Hull}\left(C\right).$$

Since *r* is arbitrary, it follows that

$$\begin{array}{c} {\left(C+C^{⊥}\right)}^{⊥}\subseteq \mathrm{Hull}\left(C\right). \end{array}$$

Conversely, for any r∈Hull(C) and any $\;a\in C,\;b\in C^{⊥}$, we have

$$\begin{array}{c} r\cdot (a+b)=r\cdot a+r\cdot b=0, \end{array}$$

Thus,

$$r\in {\left(C+C^{⊥}\right)}^{⊥},$$

which implies

$$\begin{array}{c} \mathrm{Hull}\left(C\right)\subseteq {\left(C+C^{⊥}\right)}^{⊥}. \end{array}$$

Hence,

$$\begin{array}{c} {\left(C+C^{⊥}\right)}^{⊥}=\mathrm{Hull}\left(C\right). \end{array}$$

□

**Theorem** **9** ([16,26])**.** *Let C be a linear code with parameters [n,k,d] over $F_{q}$ and e=n−k−dim(Hull(C)). Then, there exists a QECC with parameters ${\left[\left[n+e,2k-n+e,d\right]\right]}_{q}$, where $d\geq \min\{d,d\left(C+C^{⊥}\right)+1\}$.*

Using Theorems 8 and 9, we can have the following theorem.

**Theorem** **10.** *Let C be an $F_{q}R$-$\left(\lambda_{1},\lambda_{2}\right)$-constacyclic code of block length (s,t). Then, ψ(C) is a linear code with parameters [s+4t,k,d] over $F_{q}$. Set e=s+4t−k−dim(Hull(ψ(C))); then, there exists a QECC with parameters ${\left[\left[s+4t+e,2k-\left(s+4t\right)+e,d\left(Q\right)\right]\right]}_{q}$, where $d\left(Q\right)\geq \min\{d,d\left(\psi \left(C\right)+{\left(\psi \left(C\right)\right)}^{⊥}\right))+1\}$.*

**Example** **1.** *Let s=3,t=5 and $R=F_{7}\left[u_{1},u_{2},u_{3}\right]/\langle u_{i}^{2}=u_{i},u_{i}u_{j}=u_{j}u_{i}=0\rangle$, where i,j=1,2,3. In $F_{7}\left[x\right]$,*

$$x^{5}-1=\left(x-1\right)\left(x^{4}+x^{3}+x^{2}+x+1\right)$$

*and*

$$x^{3}-1=\left(x-1\right)\left(x+3\right)\left(x+5\right).$$

*Let C be a non-separable (1,1)-constacyclic code of block length (3,5) over $F_{7}R$, $g_{1}\left(x\right)=x^{4}+x^{3}+x^{2}+x+1$, $g_{2}\left(x\right)=g_{3}\left(x\right)=g_{4}\left(x\right)=x-1$. Then,*

$$C=\langle \left((x-1)(x+3),0\right),\left(\left(x+3\right),\sum_{i=1}^{4}e_{i}g_{i}\left(x\right)\right)\rangle$$

*and*

$$\begin{array}{c} \mathrm{Hull}\left(C\right)=\langle \left(\left(x-1\right)\left(x+3\right),0\right),\left(-\left(x+3\right),e_{1}\left(x^{4}+x^{3}+x^{2}+x+1\right)+\sum_{i=2}^{4}e_{i}\left(x^{5}-1\right)\right)\rangle . \end{array}$$

*By Corollary 2, we have dim(Hull(C))=2.*

*By Theorem 8, ψ(C) is a linear code over $F_{7}$ with parameters [23,14,5], and e=23−14−2=7.*

*By Lemma 10,*

$$\begin{array}{c} \psi \left(C\right)+{\left(\psi \left(C\right)\right)}^{⊥}={\left(\mathrm{Hull}\left(\psi \left(C\right)\right)\right)}^{⊥}={\left(\psi \left(\mathrm{Hull}\left(C\right)\right)\right)}^{⊥}=\psi \left({\left(\mathrm{Hull}\left(C\right)\right)}^{⊥}\right)=\psi \left(\left(\mathrm{Hull}\left(C\right)\right)\right) \end{array}$$

*and*

$$d(\psi \left(C\right)+{\left(\psi \left(C\right)\right)}^{⊥})=d\left(\mathrm{Hull}\left(C\right)\right)=7.$$

*Using Theorem 10, we can get a QECC with parameters ${\left[\left[30,12,5\right]\right]}_{7}$.*

In column 8 of Table 1, we provide several new QECCs that are not available in the database of quantum codes [29]. Among them, the first four codes have larger minimum distances than those in [29] (as shown in column 9), enabling them to detect and correct more errors; the remaining four codes are new and not available in [29]. In particular, the last code has a minimum distance greater than that of any code over $F_{25}$ in [29].

## 6. Conclusions

In this article, we extend the study of hulls of constacyclic codes from finite fields and finite rings to mixed-alphabet ring $F_{q}R$, focusing on the non-separable case. Our main algebraic contributions are as follows. We have determined the generator polynomial of the hulls of non-separable constacyclic codes over this ring, and we have derived a simplified dimension formula for these hulls. These results generalize several known results on hulls of cyclic and double cyclic codes over mixed alphabets, and they reveal how the different components of the mixed-alphabet ring interact in determining the hull structure. On the quantum coding side, it offers a new approach to constructing quantum codes with improved parameters without relying on the dual-containing condition. By applying the quantum construction X, we have obtained several new QECCs whose parameters improve upon the best known codes listed in the online quantum codes database. This shows that non-separable constacyclic codes over the mixed-alphabet ring $F_{q}R$ provide a fruitful source of QECCs.

For future research, we will explore the algebraic structure of the *l*-Galois hull of constacyclic codes over various mixed-alphabet rings, and we will construct more new QECCs with better parameters. We also plan to investigate other mixed-alphabet structures and to characterize their hulls and the resulting quantum codes. In addition, applying these hulls to fault-tolerant quantum computation schemes remains an interesting direction for further study.

## Figures and Tables

**Table 1:** New QECCs from non-separable constacyclic codes over $F_{q}R$.

*q*	(s,t)	$(\lambda_{1},\lambda_{2}$)	$\left(f_{1}\left(x\right),g_{1}\left(x\right),g_{2}\left(x\right),g_{3}\left(x\right),g_{4}\left(x\right)\right)$	ψ(C)	dim(Hull(C))	*e*	New QECCs	QECCs in Ref. [29]
2	(3,17)	(1,1)	(1,100111001,11,11,11)	[71,59,7]	1	11	${\left[\left[82,58,7\right]\right]}_{2}$	${\left[\left[82,58,6\right]\right]}_{2}$
2	(3,7)	(1,1)	(1,1,111101,11,11)	[31,23,6]	1	7	${\left[\left[38,22,6\right]\right]}_{2}$	${\left[\left[38,22,5\right]\right]}_{2}$
3	(13,7)	(−1,−1)	(1021,1212121,11,11,11)	[41,28,10]	10	3	${\left[\left[44,18,10\right]\right]}_{3}$	${\left[\left[44,18,8\right]\right]}_{3}$
7	(5,17)	(−1,−1)	(16161,16161616161616161,11,11,11)	[73,49,17]	1	23	${\left[\left[96,48,17\right]\right]}_{7}$	${\left[\left[96,48,15\right]\right]}_{7}$
13	(3,7)	(−1,−1)	(13,171,11,11,11)	[31,25,5]	2	4	${\left[\left[35,23,5\right]\right]}_{13}$	
13	(7,3)	(−1,−1)	(171,13,11,11,11)	[19,12,6]	1	6	${\left[\left[25,11,6\right]\right]}_{13}$	
13	(3,5)	(−1,−1)	(11,1(−1)1(−1)1,11,11,11)	[23,14,7]	2	7	${\left[\left[30,12,7\right]\right]}_{13}$	
$7^{2}$	(3,11)	(−1,−1)	$\left(12,1\omega^{25}66\omega^{31}1,11,11,11\right)$	[47,37,7]	7	3	${\left[\left[50,30,7\right]\right]}_{49}$	
$5^{2}$	(13,11)	(−1,−1)	$\left(1\omega^{7}1,114431,11,11,11\right)$	[57,36,8]	6	15	${\left[\left[73,30,8\right]\right]}_{25}$	

## Data Availability

Data are contained within the article.
